# A CRISPR and high-content imaging assay compliant with ACMG/AMP guidelines for clinical variant interpretation in ciliopathies

**DOI:** 10.1007/s00439-020-02228-1

**Published:** 2020-10-23

**Authors:** Liliya Nazlamova, N. Simon Thomas, Man-Kim Cheung, Jelmer Legebeke, Jenny Lord, Reuben J. Pengelly, William J. Tapper, Gabrielle Wheway

**Affiliations:** 1 Faculty of Medicine, Human Development and Health, University of Southampton, Southampton, UK; 2 Wessex Regional Genetics Laboratory, Salisbury District Hospital, Salisbury, UK; 3 Centre for Research in Biosciences, University of the West of England, Bristol, Bristol, UK; 4 University Hospital Southampton NHS Foundation Trust, Southampton, UK

## Abstract

Ciliopathies are a broad range of inherited developmental and degenerative diseases associated with structural or functional defects in motile or primary non-motile cilia. There are around 200 known ciliopathy disease genes and whilst genetic testing can provide an accurate diagnosis, 24–60% of ciliopathy patients who undergo genetic testing do not receive a genetic diagnosis. This is partly because following current guidelines from the American College of Medical Genetics and the Association for Molecular Pathology, it is difficult to provide a confident clinical diagnosis of disease caused by missense or non-coding variants, which account for more than one-third of cases of disease. Mutations in *PRPF31* are the second most common cause of the degenerative retinal ciliopathy autosomal dominant retinitis pigmentosa. Here, we present a high-throughput high-content imaging assay providing quantitative measure of effect of missense variants in *PRPF31* which meets the recently published criteria for a baseline standard in vitro test for clinical variant interpretation. This assay utilizes a new *PRPF31*^+/–^ human retinal cell line generated using CRISPR gene editing to provide a stable cell line with significantly fewer cilia in which novel missense variants are expressed and characterised. We show that high-content imaging of cells expressing missense variants in a ciliopathy gene on a null background can allow characterisation of variants according to the cilia phenotype. We hope that this will be a useful tool for clinical characterisation of *PRPF31* variants of uncertain significance, and can be extended to variant classification in other ciliopathies.

**Electronic supplementary material:**

The online version of this article (10.1007/s00439-020-02228-1) contains supplementary material, which is available to authorized users.

## Background

Ciliopathies are a broad range of inherited developmental and degenerative diseases associated with structural or functional defects in motile or primary non-motile cilia (Oud et al. 2017). Motile ciliopathies, such as primary ciliary dyskinesia, commonly present with severe respiratory problems and *situs* defects. Primary non-motile ciliopathies include both syndromic multi-organ conditions, such as Joubert syndrome and Alström syndrome, as well as single-organ disorders such as polycystic kidney disease and some forms of retinitis pigmentosa and Leber congenital amaurosis which only affect the retina. Common clinical features of these non-motile ciliopathies include retinal degeneration and kidney disease; around one-third of all the cases of retinal dystrophy can be considered retinal ciliopathies, arising as a result of defects in the photoreceptor cilium. Whilst individually rare, collectively, ciliopathies are estimated to affect ~ 1:1000 people in the general population worldwide, affecting ~ 67,500 people in the UK (Wheway et al. 2019a). However, this is likely to be an underestimate, as ciliopathies are likely to be under-diagnosed.

Ciliopathies are genetic, mostly autosomal recessive, conditions. There are ~ 200 known ciliopathy disease genes and it is expected that there are many more unidentified. Genetic testing can provide an accurate diagnosis, but 24–60% of ciliopathy patients who undergo genetic testing do not receive a genetic diagnosis (Bachmann-Gagescu et al. 2015; Knopp et al. 2015; Sawyer et al. 2016; Watson et al. 2016). This is at least in part due to the fact that following current guidelines from the American College of Medical Genetics (ACMG) and the Association for Molecular Pathology (AMP) (Richards et al. 2015), missense or non-coding variants, which account for more than one-third of cases of disease, can be challenging to interpret due to the lines of evidence that can be applied. It is estimated that around 10% of ciliopathy patients in the UK have plausibly pathogenic missense mutations in known disease genes which cannot be classified as pathogenic following current ACMG/AMP guidelines because they lack sufficient supporting evidence (e.g. segregation, recurrence, and splicing).

In vitro functional assays can provide useful lines of evidence to support variant classification but these are often labour-intensive, and there has been a lack of clarity in ACMG/AMP guidelines as to what constitutes a valid functional assay. Variant Curation Expert Panels (VCEPs) have developed guidelines for valid functional assays for specific conditions, but these vary widely from in vitro assays, splicing assays to animal model studies (Kanavy et al. 2019). A recent publication (Brnich et al. 2019) outlines general guidelines for assessing whether in vitro assays meet baseline standard for clinical variant interpretation, stating the following criteria:

1. The disease mechanism should be understood.
2. Assays must be applicable to this disease and this disease mechanism.
3. Normal/negative/wild-type AND abnormal/positive/null controls must be used AND multiple replicates must be used.
4. Variant controls must be known benign and known pathogenic.
5. Statistical analyses must be applied to calculate the level of evidence for each variant.

To facilitate standardised application of levels of evidence, Brnich et al. (2019) provide tables for calculating odds of pathogenicity values (OddsPath), with each OddsPath equating to a corresponding level of evidence strength (supporting, moderate, strong, very strong) in keeping with the ACMG/AMP variant interpretation guidelines (Richards et al. 2015). This provides a useful framework for developing variant analysis pipelines, but the work involved in optimizing and carrying out such robust in vitro assays is often beyond the scope of diagnostic labs, which do not possess the time or resources to carry out such assays for all but the most common disease genes. It is important for academic research laboratories to work with clinical diagnostic laboratories to develop robust, reliable variant analysis pipelines which meet these criteria. This is particularly important as increasing volumes of variants of unknown clinical significance are produced by genome sequencing, which is being integrated into the UK National Health Service as a standard clinical service (Wheway and Mitchison 2019).

Recent imaging screens for genes involved in ciliogenesis have demonstrated the power of high-content imaging for analysis of cilia gene function (Kim et al. 2010, 2016; Roosing et al. 2015; Wheway et al. 2015). Disturbance of cilia gene function provides a robust binary output (presence/absence of cilia) which is highly amenable to high-throughput analysis via automated imaging and image analysis, and can provide a continuous data readout in the form of percentage of cells with a single cilium. siRNA screens for novel cilia genes and cilia regulators have been highly successful in identifying novel ciliopathy disease genes and ciliary functional modules (Kim et al. 2010, 2016; Wheway et al. 2015). The advent of CRISPR gene editing provides new opportunities for exploiting such imaging approaches for classification of variants of unknown clinical significance.

One group of retinal ciliopathies (cilia-associated diseases specifically affecting the retina) are the forms of retinitis pigmentosa (RP) associated with mutations in pre-mRNA splicing factors *PRPF3, 4, 6, 8, 31, SNRNP200, CWC27* and *RP9*. Collectively, these are the second most common cause of autosomal dominant RP. Although it remains unclear why, defects in these pre-mRNA splicing factors lead to a degenerative retinal cilia phenotype which can be observed in cells harbouring pathogenic variants in these genes in the laboratory (Wheway et al. 2015; Buskin et al. 2018; Brydon et al. 2019).

All reported variants in *PRPF3, 4, 6, 8, SNRNP200, CWC27* and *RP9* are missense mutations. Most reported variants in *PRPF31* are null variants (Martin-Merida et al. 2018; Wheway et al. 2020), but there are many missense variants in *PRPF31* in public mutation databases which are labelled ‘uncertain clinical significance’. Mutations in *PRPF31* are the most common cause of autosomal dominant RP after rhodopsin mutations, and characterisation of missense variants in this gene presents a significant challenge in providing accurate diagnosis for patients. Developing tools to provide accurate genetic diagnoses in these cases is a significant clinical priority, particularly as *PRPF31* gene therapy is in development (Brydon et al. 2019).

In this study, we use CRISPR gene editing and high-throughput imaging of ciliated cells to establish a variant analysis pipeline consistent with recommendations for application of the functional evidence PS3/BS3 criterion (PS3 = well-established functional studies show a deleterious effect, BS3 = well-established functional studies show no deleterious effect) using the ACMG/AMP sequence variant interpretation framework, for accurate clinical genetic diagnosis of missense variants in *PRPF31*. We studied all *PRPF31* missense variants currently annotated as ‘uncertain clinical significance’ in patients with retinal dystrophy/retinitis pigmentosa in the ClinVar database of variant interpretations (Landrum et al. 2014, 2016).

## Methods

### Cell culture

hTERT-RPE1 cells (ATCC CRL-4000) were cultured in DMEM/F12 (50:50 mix) + 10% FCS at 37 °C, 5% CO_2_, and split at a ratio of 1:8 once per week.

### CRISPR gene knockouts

*Streptococcus pyogenes* Cas9 (spCas9) was complexed with one of four modified single-guide RNAs (sgRNAs) targeting intron 4, exon 5 or intron 5 of *PRPF31* (Synthego) to form ribonucleoprotein complexes (RNPs). sgRNA sequences were: sgRNA1 TCTGCTCGCCCCCAGGAGCT (PAM GGG), sgRNA2 CATTGTTCTTGCACTTGTCC (PAM AGG), sgRNA3 GACGACCATGATGGTGGCAT (PAM TGG), and sgRNA4 AGGGAGGCGCCGGGCCCTAA (PAM TGG). sgRNAs had the following modifications to increase stability: 2′-*O*-methyl analogs and 3′ phosphorothioate internucleotide linkages at the first three 5′ and 3′ terminal RNA residues. RNPs were prepared in 1:6 (vol:vol) ratio (protein to modified RNA oligonucleotide) in P3 solution (supplemented) and incubated for 10 min at room temperature prior nucleofecting the cell suspension (100,000 cells/5 µl P3 reagent per reaction, Lonza protocol EA104). A proportion of bulk-edited cells were harvested for DNA extraction and PCR amplification of the relevant targeted region of *PRPF31* using OneTaq polymerase (NEB). PCR products were cleaned using ExoSAP-IT (Thermo Fisher) and Sanger sequencing was performed by Source Biosciences. Sequencing traces were analysed using inference of CRISPR edits (ICE) analysis (Synthego). Of the four gRNAs tested, indel frequencies and knockout efficiencies, as measured in bulk cell populations using ICE analysis, were as follows: guide 1:32%/29%, guide 2:43%/37%, guide 3:40%/26%, guide 4:85%/72%. Guide 4 was found to target intron 5 and so was excluded from further use. Knockout efficiency of guides 1–3 was approximately equivalent, and cells edited with guide 1 grew with the healthiest appearance under phase contrast microscopy and so were taken forward for single-cell isolation.

### Single-cell cloning

Cells were dissociated using Accutase at room temperature, counted and transferred to a conical tube. Cells were collected by centrifugation at 200 *g* and washed with sterile sort buffer (Ca & Mg free PBS, 25 mM HEPES pH 7.0, 1–2.5 mM EDTA and 0.5% BSA or 1–2% FCS). Cells were collected again and resuspended at a concentration of 5–8 × 10^6^ cells/ml. Untransfected cells were used for gating cell size on the FACS Aria cell sorter (BD) and edited cells then sorted into 150 µl DMEM/F12 + 20% FCS + 10% antibiotic and antimycotic + 10 µM Y-27632 ROCK inhibitor (STEMCELL Technologies) into each well of a 96-well plate. Resultant *PRPF31* phenotype was confirmed using PCR as described in earlier methods section. Biallelic knockouts, monoallelic knockouts and unedited cells were isolated. Monoallelic knockouts and unedited controls were taken forward for further work.

### Off-target effect prediction

Cas-OFFinder (Bae et al. 2014) was used to predict potential off-target cut sites of Cas9 guided by sgRNA1. Allowing up to 3 nucleotide mismatches of the sgRNA, 15 potential off-target sites were identified in GRCh38, including 6 in introns, 1 in 3′UTR, 1 in a non-coding exon and 2 at intron/exon boundaries. These regions were visually inspected for insertions or deletions or SNVs in RNA sequence (details below) using Integrative Genomics Viewer (Robinson et al. 2011).

### Cell fractionation

Cells were fractionated into nuclear and cytoplasmic fractions. Cells were collected by scraping into fractionation buffer (20 mM HEPES pH 7.4, 10 mM KCl, 2 mM MgCl_2_, 1 mM EDTA, 1 mM EGTA) on ice, lysed through a 27 gauge needle, on ice. The nuclear pellet was collected by centrifugation at 720 × *g*, washed and dispersed through a 25 gauge needle. The supernatant containing cytoplasm was centrifuged at 10,000 *g* to remove mitochondria and any cell debris. The dispersed nuclear pellet was collected again by centrifugation at 720 × *g*, resuspended in TBS with 0.1% SDS and sonicated to shear genomic DNA and homogenize the lysate.

### RNA extraction

RNA was extracted from fractionated samples using TRIzol Reagent (Thermo Fisher). RNA quality and concentration was measured using an RNA Nano chip on the Agilent Bioanalyser 2100. Samples with total RNA concentration ≥ 20 ng/µl, RIN ≥ 6.8 and OD 260/280 were taken forward for cDNA library preparation and sequencing.

### cDNA library preparation and sequencing

cDNA libraries were prepared using Ribo-Zero Magnetic Kit for rRNA depletion and NEBNext Ultra Directional RNA Library Prep Kit library prep kit by Novogene Inc. Library quality was assessed using a broad range DNA chip on the Agilent Bioanalyser 2100. Library concentration was assessed using Qubit and q-PCR. Libraries were pooled, and paired-end 150 bp sequencing to a depth of 20 M reads per fraction (40 M reads per sample) was performed on an Illumina HiSeq2500 by Novogene Inc.

### Raw-data quality control

Raw FASTQ reads were subjected to adapter trimming and quality filtering (reads containing *N* > 10%, reads where > 50% of read has Qscore <  = 5) by Novogene Inc.

Quality of sequence was assessed using FastQC v0.11.5 (https://www.bioinformatics.babraham.ac.uk/projects/fastqc/). No further data filtering or trimming was applied.

### Data deposition

Raw FASTQ reads after adapter trimming and quality filtering (reads containing *N* > 10%, reads where > 50% of read has Qscore <  = 5) were deposited on the Sequence Read Archive, SRA accession PRJNA622794.

### Alignment for transcript level analysis

Paired FASTQ files were aligned to GRCh38 human genome reference using GENCODE v29 gene annotations (Frankish et al. 2019) and STAR v2.6.0a splice aware aligner (Dobin et al. 2013), using ENCODE recommend options (3.2.2 in the STAR manual (https://github.com/alexdobin/STAR/blob/master/doc/STARmanual.pdf). The two-pass alignment method was used, with soft clipping activated.

### Alignment quality control and transcript level abundance estimates

BAM files sorted by chromosomal coordinates were assessed for saturation of known splice junctions and transcript abundance estimates in fragments per kilobase of exon per million reads (FPKM) were calculated using RSeqQC v3.0.1 (Wang et al. 2012).

### Differential splicing analysis

rMATs v4.0.2 (rMATS turbo) (Shen et al. 2014) was used to statistically measure differences in splicing between replicates of wild-type and mutant sequence. BAM files aligned with STAR v2.6.0a two-pass method with soft clipping suppressed were used as input.

### Protein extraction

Total protein was extracted from cells using 1% NP40 lysis buffer and scraping. Insoluble material was pelleted by centrifugation at 10,000 × *g*. Cell fractionation was carried out by scraping cells into fractionation buffer containing 1 mM DTT and passed through a syringe 10 times. Nuclei were pelleted at 720 × *g* for 5 min and separated from the cytoplasmic supernatant. Insoluble cytoplasmic material was pelleted using centrifugation at 10,000 × *g* for 5 min. Nuclei were washed, and lysed with 0.1% SDS and sonication. Insoluble nuclear material was pelleted using centrifugation at 10,000 × *g* for 5 min.

### SDS-PAGE and western blotting

20 µg of total protein per sample with 2 × SDS loading buffer was loaded onto pre-cast 4–12% Bis–Tris gels (Life Technologies) alongside Spectra Multicolor Broad range Protein ladder (Thermo Fisher). Samples were separated by electrophoresis. Protein was transferred to PVDF membrane. Membranes were incubated with blocking solution [5% (w/v) non-fat milk/PBS], and incubated with primary antibody overnight at 4 °C. After washing, membranes were incubated with secondary antibody for 1 h at room temperature and exposed using 680-nm and/or 780-nm laser (LiCor Odyssey et al.), or incubated with SuperSignal West Femto reagent (Pierce) and exposed using Chemiluminescence settings on ChemiDoc MP imaging system (Bio-Rad).

### Primary antibodies for WB

Mouse anti-ß actin clone AC-15. 1:4000. Sigma-Aldrich A1978

Mouse anti-c myc 1:5000 (Sigma)

Rabbit anti-PRPF31 primary antibody 1:1000 (AbCam)

Rabbit anti-PRPF6 primary antibody 1:1000 (Proteintech)

### Secondary antibodies for WB

Donkey anti-mouse 680 1:20,000 (LiCor)

Donkey anti-rabbit 800 1:20,000 (LiCor)

Donkey anti-mouse HRP (Dako)

Donkey anti-rabbit HRP (Dako)

### Variant classification

We extracted all *PRPF31* missense variants annotated as ‘uncertain clinical significance’ in patients with retinal dystrophy/retinitis pigmentosa in ClinVar (26 variants). Total number of reported cases with the same phenotype for each variant were identified from PubMed and HGMDPro searches. Protein functional effect was predicted using 3 in silico tools in Alamut Visual 2.4 (Interactive Biosoftware); Align GVGD (Mathe et al. 2006; Tavtigian et al. 2006), SIFT (Ng and Henikoff 2003) and PolyPhen 2 (Adzhubei et al. 2013). The location of the mutated residue in relation to functional domains was identified using previously published analysis of the structure of PRPF31 (Wheway et al. 2020). The effect of variant on splicing was predicted using the Splicing Prediction Module in Alamut Visual 2.4 (Interactive Biosoftware) which aggregates 5 tools; SpliceSiteFinder-like, MaxEntScan (Yeo and Burge 2004), NNSPLICE (Reese et al. 1997), GeneSplicer (Pertea et al. 2001) and Human Splicing Finder (Desmet et al. 2009). Other changes at the same codon/nucleotide were recorded where these were found in GnomAD v3. Population frequency of allele was extracted from GnomAD v3 (overall minor allele frequency (MAF) of all ethnic groups). We set a MAF cutoff of 2.3 × 10^–5^ based on the calculation (1/3000*0.25*0.055)/2 where 1/3000 is the prevalence of RP (Golovleva et al. 2010; Sharon and Banin 2015), 0.25 is the proportion of RP which is autosomal dominant (Daiger et al. 2014) and 0.055 is the fraction of adRP due to sequence variants in *PRPF31* (Sullivan et al. 2006, 2013), division by 2 assumes that one single variant is causing disease, and final result is adjusted by tenfold to account for incomplete penetrance seen in this condition. These lines of evidence were used to apply PVS (very strong evidence of pathogenicity)/PS (strong evidence of pathogenicity)/PM (moderate evidence of pathogenicity)/PP (supporting evidence of pathogenicity)/BA (standalone evidence of benign impact)/BS (strong evidence of benign impact)/BP (supporting evidence of benign impact) criteria to classify each variant following ACMG/AMP guidelines.

### Variant construct cloning

Full-length, sequence-validated *PRPF31* ORF clone with C-terminal myc tag was obtained from Origene. Single-nucleotide variants were introduced using NEB Q5 site-directed mutagenesis kit. The entire wild-type and mutant clone sequence was verified by Sanger sequencing (Source Bioscience).

### Cell transfection

The construct was transfected into hTERT-RPE1 cells using the Lonza 4D Nucleofector. Construct was mixed with P3 solution (supplemented) and incubated for 10 min at room temperature prior to nucleofecting the cell suspension (100,000 cells/5 µl P3 reagent per reaction, Lonza protocol EA104).

### Imaging plate setup

20 µl of nucleofected cells were plated at a density of 1 × 10^5^ cells ml^−1^ into 80 µl complete media per well in 96-well optical bottom Perkin Elmer ViewPlates. The outer wells were filled with media without cells to reduce edge effects. Cells were cultured for 48 h before media was changed to serum-free media. Cells were fixed 24 h later.

### Immunocytochemistry of imaging plates

Wells were emptied by inversion of plates, and washed with warm Dulbecco’s PBS (Sigma). DPBS was removed by plate inversion and cells were fixed with ice-cold methanol for 5 min at − 80 °C. Methanol was removed by plate inversion and cells were washed twice with PBS and non-specific antibody-binding sites blocked with 1% non-fat milk powder/PBS (w/v) for 15 min at room temperature. Cells were incubated with primary antibodies in blocking solution for 1 h at room temperature and secondary antibodies + DAPI for 1 h at room temperature in the dark. Mowiol was added to wells, and plates stored until imaging.

### Primary antibodies for immunocytochemistry

Rabbit anti-ARL13B primary antibody 1:200 (Proteintech)

Mouse anti-c myc 1:1000 (Sigma)

### Secondary antibodies for immunocytochemistry

Donkey anti-mouse IgG AlexaFluor 488 1:500 (ThermoFisher)

Donkey anti-goat IgG AlexaFluor 568 1:500 (ThermoFisher)

### High-throughput confocal imaging

Imaging was carried out on a Perkin Elmer Opera LX with 20 × and 60 × water immersion lenses at Wolfson Bioimaging Centre, University of Bristol.

### Image analysis

Image analysis was performed using custom scripts optimised on CellProfiler (Carpenter et al. 2006). Analysis included nuclear recognition and counting, cell recognition, exclusion of border objects and counting of whole cells, cilia recognition and counting, and quantification of the percentage of whole cells with a single cilium. Analysis scripts are freely available for re-use and modification under a GNU licence from https://github.com/GWheway/cilia_HCI. Median and median absolute deviation of mock transfected cells were used to calculate robust *z* scores (Zhang 2007; Chung et al. 2008; Birmingham et al. 2009) of cell number and percentage of whole cells with a single cilium in transfected cells.

## Results

### Production and characterisation of *PRPF31* knockout (KO) retinal pigment epithelium (RPE1) cell line

**Fig. 1:**
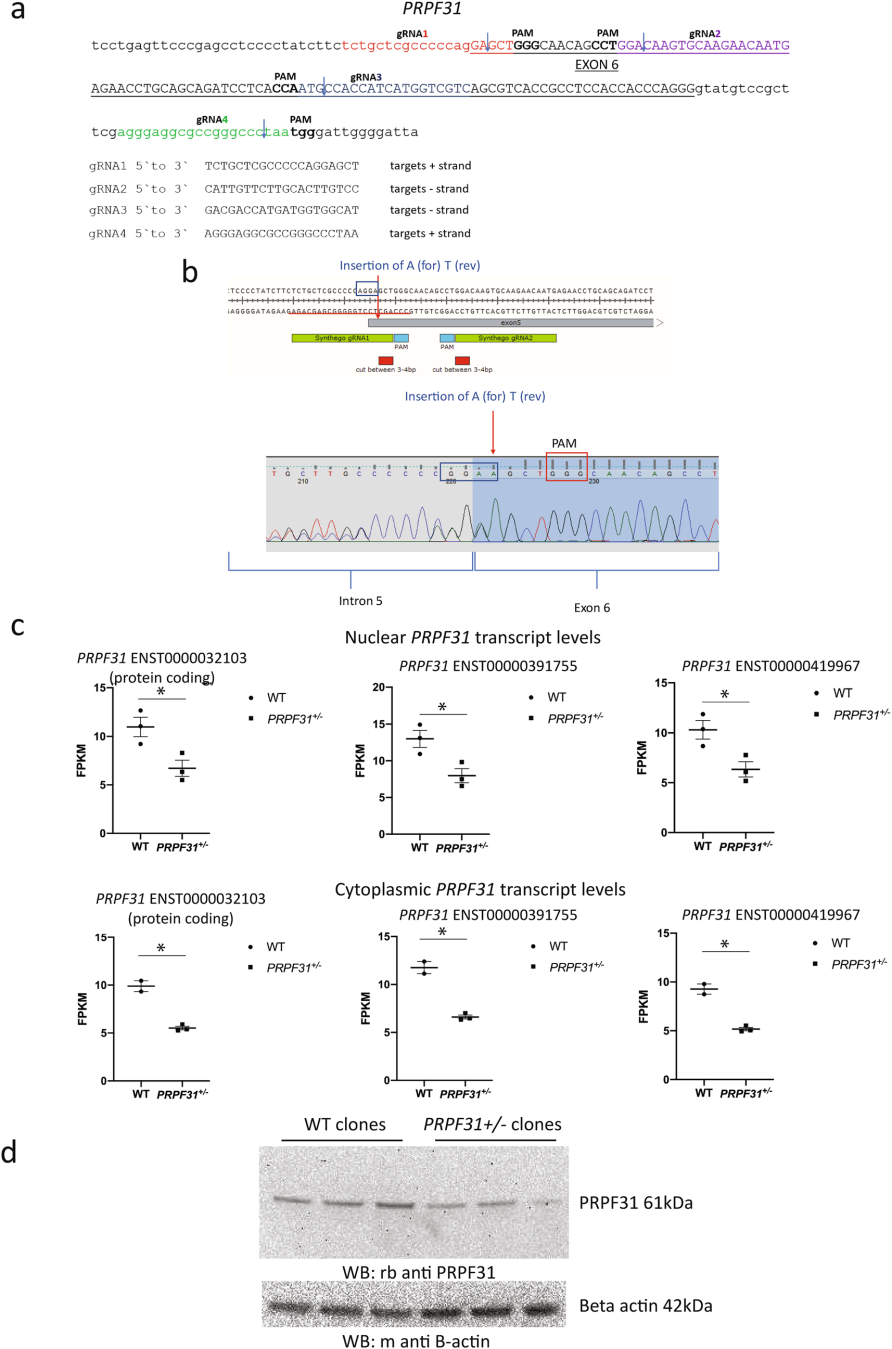
Heterozygous knockout of *PRPF31* in hTERT-RPE1 cells by insertion of single nucleotide in exon 6 by CRISPR/Cas9 editing. **a** Mapping of single-guide RNAs to *PRPF31* exon 6 (coding exon 5) used in CRISPR editing approach. **b** Schematic diagram and electropherogram sequence trace showing heterozygous insertion of A near intron 5/exon 6 boundary of *PRPF31* in hTERT-RPE1 cells. **c** Scatterplots showing roughly 50% reduction in three major *PRPF31* transcripts in edited cells compared to wild-type cells (FPKM = fragments per kilobase of transcript per million mapped reads).*  *p* < 0.05 two-sample *t* test. Individual FPKM values for each cell clone are shown, along with mean and standard error of the mean. **d** Western blot showing reduced expression of PRPF31 protein (top) relative to beta-actin control expression (bottom) in three independent *PRPF31*^+/–^ edited clones compared to three independent wild-type non-edited sister clones. PRPF31 blot used AbCam rabbit anti-PRPF31 antibody

It remains unclear whether missense variants in *PRPF31* cause disease by dominant negative effects or haploinsufficiency. It has been suggested that *PRPF31*-associated disease is caused by a combined dominant negative and haploinsufficiency mechanism (Rose and Bhattacharya 2016; Wheway et al. 2020). To produce a disease-relevant human cell model which would allow analysis of PRPF31 variants acting via a mechanism of dominant negative effects or haploinsufficiency, we produced stable monoclonal *PRPF31* heterozygous mutant retinal pigment epithelium (RPE1) cell lines. We achieved this using purified wild-type Cas9 and four single-guide RNAs targeting intron 5 and exon 6 (coding exon 5) of *PRPF31* which were modified to increase stability (Fig. 1a). We achieved up to 85% indel frequency, with up to 72% overall knockout efficiency. From the pool of edited cells from sgRNA1, we used single-cell sorting to isolate clones of *PRPF31*^+/–^ cells with heterozygous knockouts and wild-type unedited sister clones. We took three of each on for further analysis. In all the three heterozygous clones, we confirmed insertion of A at the intron 5/exon 6 boundary of *PRPF31* NC_000019.10:g.54123455_54123456insA (NM_015629.4:c.422_423insA) (p.Glu141fs) which causes a frameshift and premature termination codon (Fig. 1b). We performed whole transcriptome sequencing on RNA from the nucleus (a mixture of completely and incompletely spliced transcripts) and cytoplasm (only completely spliced transcripts) from all 6 clones (SRA accession PRJNA622794). We analysed predicted off-target changes in each clone through manual analysis of target regions in our RNAseq data in IGV, via analysis of differential gene expression using the edgeR package (Robinson et al. 2010; McCarthy et al. 2012) and analysis of differential splicing using rMATS turbo (Shen et al. 2014) (Supplementary Table 2). We found no evidence of sequence changes or expression changes in any of the genes predicted to be off-target sites (with 3 mismatches) but found statistically significant differential usage of 3 exons in *MEGF6* between wild-type and mutant clones. Exons 25 (ENSE00001477187) and 24 (ENSE00001477188) of ENST00000356575.9, and exon 27 (ENSE00001308186) of ENST00000294599.8 are each significantly skipped in mutants, FDR p value = 0.0279, 0.0343 and 0.0086, respectively) (Supplementary Table 2). *MEGF6* is a poorly characterised protein which has not been linked to cilia, and we do not expect this change to affect our cell phenotype, but it is important to note this splicing variation in a gene which could potentially be an off-target effect of our CRISPR guide RNAs. Analysis of splicing patterns of *PRPF31* showed no significant change in splicing of intron 5 or exon 6 in the mutant clones compare to wild-type (no differential 3′ splice site usage, skipping of exon 6 or retention of intron 5–6). However, we did unexpectedly observe an increase of retention of intron 12–13 in the nuclear fraction of the mutant cells (FDR *p* value = 0.0141 when considering only reads mapping splice junctions, or FDR *p* value = 0.0091 when also considering reads mapping to the intron), although this was not observed in the cytoplasmic fraction of the cells (Supplementary Fig. 1). We hypothesise that mutant *PRPF31* may experience changes in the dynamic of splicing, with less efficient removal of introns before export from the nucleus.

Transcript level expression analysis of RNA sequence data showed expression of three *PRPF31* transcripts in both mutant and wild-type cell lines; ENST00000419967.5, ENST00000391755.1 and protein-coding ENST00000321030.8, with an approximately 50% reduction in all *PRPF31* transcripts in the mutant clones (Fig. 1c). Analysis of reads around the CRISPR insertion site (i.e. at the intron 5/exon 6 boundary) in the mutant clones showed that very few reads contained the insertion. In nuclear RNA from the mutant clones, the ratio of wild-type reads to reads with the insertion was 46:2 (4.2% insertion), 92:11 (10.7% insertion) and 48:0 (0% insertion). Roughly, the same proportions of reads with insert were seen in the cytoplasmic RNA from mutant clones (70:2, 61:4, 53:2, i.e. 2.8%, 6.2%, and 3.6%). This suggests that *PRPF31* is preferentially expressed from the wild-type allele in the mutant cells, and both wild-type and mutant transcripts are exported to the cytoplasm. If the differences in transcript abundance were due to nonsense-mediated decay (NMD) of the mutant transcript, we would expect to see approximately equal amounts of the wild-type and mutant transcripts in the nucleus, but a reduction of mutant transcript in the cytoplasm where NMD occurs. This suggests that in this cell model the disease phenotypes (see later) are caused by haploinsufficiency. Indeed, western blotting of protein extracts from wild-type and mutant clones confirmed reduction in PRPF31 protein levels in mutant cells compared to wild-type control cells with no detectable expression of any mutant protein (Fig. 1d).

**Fig. 2:**
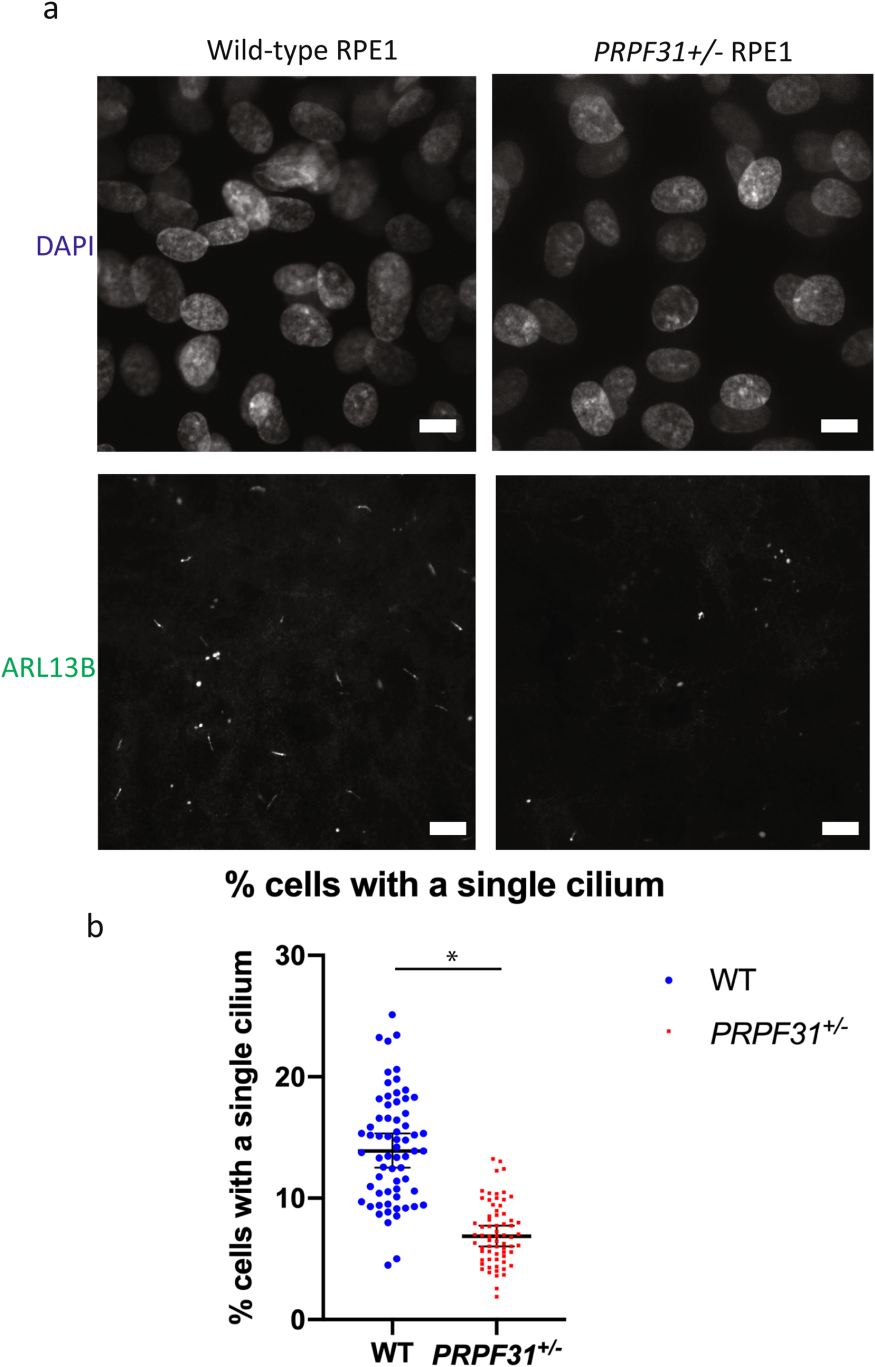
Cellular phenotype of wild-type and *PRPF31*^+/–^ mutant RPE1 clones. **a** Higher magnification images of wild-type and *PRPF31*^+/–^ mutant RPE1 clone showing nuclear DAPI stain and cilia immunostained with ARL13B and 488 nm secondary antibody. The lower rate of ciliation can be seen in the *PRPF31*^+/–^ mutant RPE1 clone. Scale bar = 10 µm. **b** Scatterplot showing individual data points for measurement of percentage cells with a single cilium in wild-type and *PRPF31*^+/–^ mutant RPE1 clones. Each datapoint represents one field of view in 1-well of a 96-well plate. Median and 95% confidence interval are shown

As has been previously reported, mutation of *PRPF31* is associated with reduction in the number and length of primary cilia on multiple cell types (Wheway et al. 2015, Buskin et al. 2018, Wheway et al. 2019a, b). To investigate whether this phenotype was observed in our mutant clones in an unbiased way, we developed a high-throughput imaging and automated image analysis workflow (Supplementary Fig. 2) to quantify number of cilia in mutant cells compared to wild-type cells (Fig. 2a). We also assayed a range of other phenotypes which have been reported in *PRPF31* mutants, including cell number, number of micronuclei per cell, nuclear area, nuclear shape (compactness, eccentricity) and nuclei staining intensity. Whilst these assays showed a general trend in reduced cell number, increased number of micronuclei per cell and reduced nuclear area in mutant clones compared to wild-type clones, the most robust and consistent phenotype we observed was the loss of cilia phenotype in *PRPF31*^*+/–*^ =cells (Fig. 2b).

### Classification of missense variants in *PRPF31* following ACMG/AMP guidelines and selection of variants to test in vitro

Of the 24 missense variants in *PRPF31* labelled ‘uncertain significance’ in patients with RP or retinal dystrophy in ClinVar, our assessment following ACMG/AMG guidelines confirmed that all are VUS (Table 1). We selected five variants at random to test in vitro:

**Table 1:**
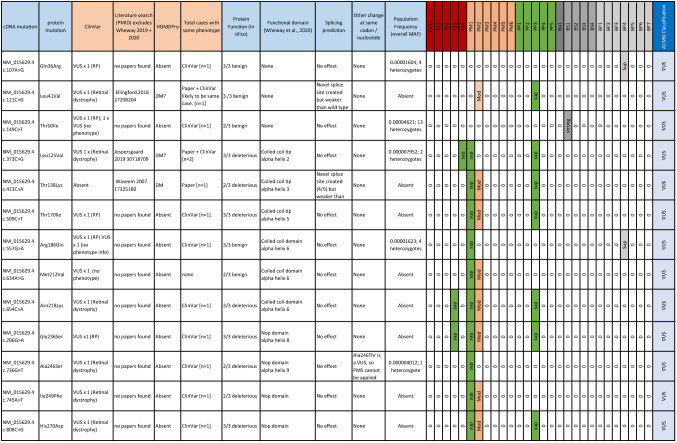

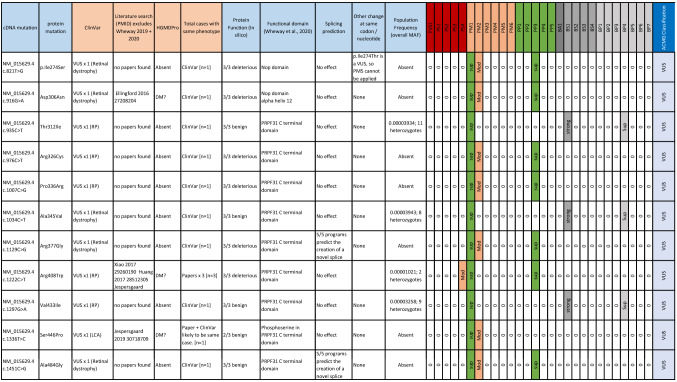
Summary of ClinVar missense variants of uncertain clinical significance in *PRPF31*

Lines of evidence and ACMG/AMP classification of all *PRPF31* missense changes deposited in ClinVar as variants of ‘uncertain clinical significance’ in patients with retinitis pigmentosa or retinal dystrophy. The table summarises location and effect on cDNA and protein, number of reported cases, functional effect predicted by Align GVGD, SIFT and PolyPhen 2, functional domain of variant, effect on splicing predicted by Splicing Prediction Module in Alamut Visual 2.4 (SpliceSiteFinder-like, MaxEntScan, NNSPLICE, GeneSplicer and Human Splicing Finder, any other reported variants in this amino acid, population frequency from GnomAD v3 and whether PVS (very strong evidence of pathogenicity)/PS (strong evidence of pathogenicity)/PM (moderate evidence of pathogenicity)/PP (supporting evidence of pathogenicity)/BA (standalone evidence of benign impact)/BS (strong evidence of benign impact)/BP (supporting evidence of benign impact) criteria can be applied according to ACMG/AMP guidelines, and overall variant classification. ACMG lines of evidence are taken from Richards et al (2015). Very strong and strong lines of pathogenic evidence are indicated in red, moderate lines of evidence in buff and supporting lines of evidence in green. Lines of benign evidence are shown in grey. The colour indicates the strength at which a line of evidence has been applied. For example, a moderate line of pathogenic evidence that has been downgraded to supporting will be shown in green

*VUS *variant of uncertain significance

*PRPF31* c.149C > T p.Thr50Ile

*PRPF31* c.413C > A p.Thr138Lys

*PRPF31* c.634A > G p.Met212Val

*PRPF31* c.736G > A p.Ala246Thr

*PRPF31* c.1297G > A p.Val433Ile

### Selection of control variants

The Brnich et al (2019) paper describes two types of controls in in vitro variant assays; ‘experimental controls’ which ‘demonstrate the dynamic range of the assay (e.g. the readout of the assay with wild type and null effect) and ‘clinical validation controls’ of known pathogenic and known benign variants. We selected the following controls:

#### Experimental controls

Wild-type (WT) *PRPF31*-positive control

Empty vector negative control

#### Validation controls

##### Benign controls

We selected the three most common exonic variants in *PRPF31* in control population database GnomAD as benign variant validation control (Table 2). We discovered upon sequencing, our PRPF31 expression clone that it already contained c.735C > T and c.1467C > T, so instead we edited these back to c.735T > C and c.1467T > C using site-directed mutagenesis and used these as two of our benign variants, so our benign validation controls were:

**Table 2:** Coding variants in PRPF31 with the highest allele frequency in GnomAD 3 database

GRCh38 variant	rsID	Transcript mutation	Protein mutation	Allele count	Allele number	Allele frequency	Homozygote count
19: 54124536C > T	rs11556769	c.735C > T	p.Pro245Pro	12,247	143,186	0.0855321	567
19: 54131399C > T	rs62144168	c.1467C > T	p.Val489Val	11,777	143,324	0.0821705	509
19: 54123785C > T	rs1058572	c.564G > A	p.Glu188Glu	3210	143,298	0.0224009	55

GRCh38 chromosomal coordinates, rsID, effect on cDNA, effect on protein, allele count, allele number, allele frequency and homozygote count across all populations in GnomAD of the 3 most common PRPF31-coding variants, which we used as benign controls in our assay

*PRPF31* c.564G > A p.Glu188Glu

*PRPF31* c.735T > C p.Pro245Pro

*PRPF31* c.1467T > C p.Val489Val

##### Pathogenic controls

We selected the three *PRPF31* missense mutatinso which have previously been published as pathogenic with characterisation by in vitro experiments as pathogenic validation controls.

*PRPF31* c.341T > A p.Ile114Asn (Wheway et al. 2019b)

*PRPF31* c.581C > A p.Ala194Glu (Deery et al. 2002)

*PRPF31* c.646G > C p.Ala216Pro (Deery et al. 2002)

### Characterisation of PRPF31 missense variants using high-throughput imaging

We transfected PRPF31^+/–^ cells with plasmid constructs expressing full-length human PRPF31 with a myc-DDK tag, under the control of a CMV promoter, with the control or test missense mutations introduced by site-directed mutagenesis to investigate their ability to restore cilia growth in the mutant cell line.

To satisfy the requirements of Brnich et al. we included multiple technical replicates of each construct per plate (3) and repeated each experimental plate in 2–4 independent biological replicates. *PRPF31*^+/–^ clone 21 was used for two plates, and *PRPF31*^+/–^ clone 18 was used for two plates. In each well, six fields of view were imaged. In each well, the median % cells with a single cilium was measured, and robust *z* score calculated, comparing this median to the median and median absolute deviation of mock transfected cells (Huang da et al. 2009). The robust *z* score is a measure of the difference between the median of three technical replicates on one plate (the three wells containing a specific construct) and the median of the three technical replicates of the negative control on the same plate (the three wells containing mock transfected cells), normalised by the median absolute deviation of the negative control population in this plate ($\mathrm{robust} z=\frac{x-\mathrm{median}}{\mathrm{MAD}})$. This provides a relative and normalised score of change in ciliation compared to the negative control population (mock transfected cells) on a per-plate basis, allowing comparisons between different biological replicates. The robust *z* score metric is used rather than the *z* score because it more robust to outliers than the *z* score, and is thus useful for high-throughput high-content imaging assays which involve a large number of image captures and image analyses.

**Fig. 3:**
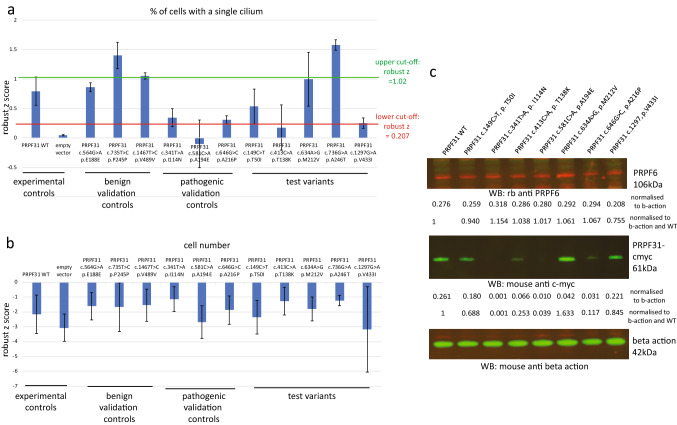
High-throughput screening of the effect of expression of specific PRPF31 variants in *PRPF31*^+/–^ mutant clones. **a** Bar graph showing the effect of expression of specific *PRPF31* variants on the percentage of cells with a single cilium in *PRPF31*^+/–^ mutant clones. Data plotted is the mean robust *z* score of *n* = 3 technical replicates across *n* = 2 or 4 independent biological replicates. Error bars show standard error of the mean. The red line marks the mean robust *z* score of all pathogenic controls, setting the lower threshold below which a novel test variant can be considered pathogenic. The green line marks the mean robust *z* score of all benign controls, setting the upper threshold above which a novel test variant can be considered benign. Results between these values should be considered indeterminate. **b** Bar graph showing effect of expression of specific PRPF31 variants on cell number in *PRPF31*^+/–^ mutant clones. Data plotted is the mean robust *z* score of *n* = 3 technical replicates across *n* = 2 or 4 independent biological replicates. Error bars show standard error of the mean. **c** Western blot showing level of expression of soluble PRPF6 (top), PRPF31 (middle) and beta-actin loading control (bottom). Intensity of bands are expressed normalised to beta-actin loading control and wild-type control

Transfection with the experimental control vectors confirmed the effect of the wild-type *PRPF31* which rescued the loss of cilia phenotype (mean robust *z* = 0.792), and the effect of empty vector transfection which did not rescue the loss of cilia phenotype (mean robust *z* = 0.049). The three benign validation controls (*PRPF31* c.564G > A p.Glu188Glu, c.735T > C p.Pro245Pro, c.1467T > C p.Val489Val) rescued the loss of cilia phenotypes with different levels of effectiveness, with a mean robust *z* score of 1.10. The mean robust *z* score of all benign controls and wild-type transfection control was 1.02. This allows an upper cutoff robust *z* score of 1.02 to be set, so that any variant construct which rescues ciliation to a greater degree than this can be considered benign in this assay. None of the three pathogenic validation controls (*PRPF31* c.341T > A p.Ile114Asn, c.581C > A p.Ala194Glu, c.646G > C p.Ala216Pro) rescued cilia at a rate comparable with the benign controls (Fig. 3a). The most severe pathogenic control mutation was *PRPF31* c.581C > A p.Ala194Glu which actually reduced the percentage of cells with a single cilium in the mutant cell line (Fig. 3a). c.646G > C p.Ala216Pro rescued ciliogenesis more than the other two pathogenic validation controls, suggestion that this is a less severe missense mutation. The mean robust *z* score of all pathogenic validation controls was 0.207. This allows a lower cutoff robust *z* score of 0.207 to be set, so that any variant construct which rescues ciliation to a lesser degree than this can be considered pathogenic in this assay. Any variant construct which has an effect on ciliation between 0.207 and 1.10 robust *z* should be considered indeterminate. According to the recommendations for application of the functional evidence PS3/BS3 criterion using the ACMG/AMP sequence variant interpretation framework, with three benign validation controls and three pathogenic validation controls this assay allows BS3_supporting to be applied to variants which rescue ciliogenesis with robust *z* score > 1.10 in this assay, and PS3_supporting evidence to be applied to variants which rescue ciliogenesis with robust *z* score < 0.207 in this assay.

Using the above criteria, of the novel missenses being tested, *PRPF31* c.736G > A p.Ala246Thr could have BS3_supporting applied and *PRPF31* c.413C > A p.Thr138Lys could have PS3_supporting evidence applied to the lines of evidence for classification of these variants (Fig. 3a). A study of cell number showed that transfection caused a reduction in cell number, and several of the variants which failed to rescue ciliogenesis (*PRPF31* c.581C > A p.Ala194Glu and *PRPF31* c.1297G > A p.Val433Ile) also showed a further modest reduction in cell number (Fig. 3b). However, overall, there was no clear correlation between severity of effect on cilia phenotype and effect on cell number.

To confirm PRPF31 protein expression from constructs which did not show rescue of ciliogenesis, we extracted protein from transfected cells and analysed expression levels by western blotting. Densitometry analysis of c-myc bands normalised to B-actin control bands (both normalised to total background intensity) showed that constructs were expressed but some missense-mutated forms of *PRPF31* (c.581C > A p.Ala194Glu and c.646G > C p.Ala216Pro) were associated with reduced stability and solubility of the protein, appearing as lower levels in the soluble fraction of cell extracts (Fig. 3c). We have previously reported that c.341T > A p.Ile114Asn shows complete instability and insolubility of mutant protein (Wheway et al. 2019a, b) and we infer that this accounts for the lack of observable p.Ile114Asn protein on the western blot (Fig. 3c). 3D structural analysis predicts that c.149C > T pT50I would interfere with binding to PRPF6. We did see a small decrease in total level of PRPF6 in cell transfected with this construct (Fig. 3c) but we did not investigate PRPF31/PRPF6 interactions.

## Discussion

Here, we present a high-throughput high-content imaging assay providing quantitative measure of effect of missense variants in the second most common cause of autosomal dominant RP, *PRPF31*. Our screening assay meets the criteria for a baseline standard in vitro test for clinical variant interpretation (Brnich et al. 2019) because the disease mechanism is understood (combined haploinsufficiency/dominant negative effects), the assay is applicable to this disease and this disease mechanism, normal/negative/wild-type and abnormal/positive/null controls are used on each assay plate, multiple replicates are used (each variant and control in 3 wells per plate, each plate repeated at least twice), variant controls are known benign and known pathogenic, and statistical analysis has been applied to calculate the level of evidence for each variant (robust *z* scores, OddsPath). This assay utilizes a new and well-characterised *PRPF31*^+/–^ human retinal cell line generated using CRISPR gene editing. The mutant cells have significantly fewer cilia than wild-type cells, allowing rescue of ciliogenesis with benign or mild variants, but do not totally lack cilia, so loss of cilia effects can be observed.

The results of the assay provide BS3_supporting evidence to the classification of novel uncharacterised *PRPF31* variant *PRPF31* c.736G > A p.Ala246Thr and PS3_supporting evidence to the classification of novel uncharacterised *PRPF31* variant *PRPF31* c.413C > A p.Thr138Lys which, in combination with other evidence, can allow a sequence variant to be classified as pathogenic, likely pathogenic, benign or likely benign (Richards et al. 2015). In the case of these two variants, the additional supporting evidence provided by this in vitro assay did not change the variant classifications, but in addition to other evidence in a clinical setting in which more is known about the patients with these variants this supporting evidence may support characterisation of the variants as (likely) pathogenic or (likely) benign.

Providing in vitro evidence to aid classification of clinical variants is of significant importance to allow accurate genetic diagnoses to be made, to enable targeted testing of other family members, aid family planning, allow pre-implantation diagnosis and inform eligibility for gene therapy trials. With *PRPF31* gene therapy in development, there is an urgent need for tools for accurate molecular diagnosis (Brydon et al. 2019).

The imaging-based screen uses a simple and robust image analysis algorithm to test a consistent cellular phenotype observed in *PRPF31* mutant cells; reduction in the number of cells with a single cilium. The assay provides a continuous data readout in the form of percentage of cells with a single cilium, which has the potential to provide more than a simple binary readout of pathogenic/benign but a measure of the extent of pathogenicity of each variant. The findings of this assay and other such assays can also provide novel insights into disease mechanism and prognosis. Although data relating to genotype–phenotype correlations in cases of patients with missense variants in *PRPF31* is sparse (Wheway et al. 2020), we hypothesise that the variants with the most significant effect on cilia will be associated with the earliest onset and worst prognosis.

## Conclusion

High-content imaging assays of ciliated cells can be adapted to meet baseline standard criteria for in vitro assays for characterisation of variants of uncertain clinical significance in human ciliopathies. Cells expressing missense variants in a ciliopathy gene on a null background can allow characterisation of variants according to the cilia phenotype. We hope that this will be a useful tool for clinical characterisation of *PRPF31* variants of uncertain significance, and can be extended to variant classification in other ciliopathies.

## Electronic supplementary material

Below is the link to the electronic supplementary material.

Supplementary file1 Supplementary Figure 1 – Differential splicing of PRPF31 intron 12-13 in wild-type and PRPF31+/- mutant clones. (a) Sashimi plot showing statistically significantly lower levels of splicing of intron 12-13 in the nuclear RNA of PRPF31+/- clones compared to wild-type clones. (b) rMATS statistical analysis of this differential splicing in nucleus, showing intron inclusion level for wild-type and mutant clones, intron inclusion level difference and p values with a without correction for false discovery rate (FDR). (c) Sashimi plot showing no statistically significantly different level of inclusion of intron 12-13 in the cytoplasmic RNA of PRPF31+/- clones compared to wild-type clones. (d) rMATS statistical analysis of this differential splicing in cytoplasm, showing intron inclusion level for wild-type and mutant clones, intron inclusion level difference and p values with a without correction for false discovery rate (FDR). SJ = only reads mapping to splice junctions considered SJ + I = reads mapping to splice junctions and to intron considered. (PDF 98 kb)

Supplementary file2 Supplementary Figure 2 – High content image analysis workflow of nuclei and cilia in wild-type and PRPF31+/- mutant clones. (a) Top two rows of panels show DAPI stained nuclei and ARL13B antibody-stained cilia from wild-type and mutant cells in raw output images from Opera confocal high-throughput imager. Lower four panels show automated image analysis using CellProfiler. Insets show magnified images from each panel. (PDF 1745 kb)

Supplementary file3 Supplementary Table 1. Table showing sequence of sgRNA1 which was used to introduce CRISPR indel, genomic DNA sequence of potential off-target mapping sites, with 3 mismatches allowed (mismatches shown in lower case), chromosomal location of these potential off-target sites, whether they are on the + or – strand, number of mismatches, gene name and feature targeted. (XLSX 10 kb)

Supplementary file4 Supplementary Table 2. Summary of analysis of potential off-target CRISPR cut sites, showing findings observed in IGV, through differential gene expression analysis by edgeR, and differential splicing analysis by rMATS, including alternative 3’ splice site usage (A3SS), alternative 5’ splice site usage (A5SS), mutually exclusive exons (MXE), retained introns (RI) and spliced exons (SE). (XLSX 12 kb)

## Data Availability

Raw FASTQ reads after adapter trimming and quality filtering (reads containing *N* > 10%, reads where > 50% of read has Qscore <  = 5) are available for download from the Sequence Read Archive, SRA accession PRJNA622794. *PRPF31*^*+/–*^ hTERT-RPE1 clonal cell lines are available on request from the lab of Dr Gabrielle Wheway. Analysis scripts are freely available for re-use and modification under a GNU licence from https://github.com/GWheway/cilia_HCI.
