# Supplementary material for: A CRISPR and high-content imaging assay compliant with ACMG/AMP guidelines for clinical variant interpretation in ciliopathies
Source: Hum Genet. 2020 Oct 23;140(4):593–607. doi: 10.1007/s00439-020-02228-1 (PMC7981318; doi:10.1007/s00439-020-02228-1)
Supplement: Supplementary file 1 — Supplementary file1 Supplementary Figure 1 – Differential splicing of PRPF31 intron 12-13 in wild-type and PRPF31+/- mutant clones. (a) Sashimi plot showing statistically significantly lower levels of splicing of intron 12-13 in the nuclear RNA of PRPF31+/- clones compared to wild-type clones. (b) rMATS statistical analysis of this differential splicing in nucleus, showing intron inclusion level for wild-type and mutant clones, intron inclusion level difference and p values with a without correction for false discovery rate (FDR). (c) Sashimi plot showing no statistically significantly different level of inclusion of intron 12-13 in the cytoplasmic RNA of PRPF31+/- clones compared to wild-type clones. (d) rMATS statistical analysis of this differential splicing in cytoplasm, showing intron inclusion level for wild-type and mutant clones, intron inclusion level difference and p values with a without correction for false discovery rate (FDR). SJ = only reads mapping to splice junctions considered SJ + I = reads mapping to splice junctions and to intron considered. (PDF 98 kb) [file 439_2020_2228_MOESM1_ESM.pdf]

a

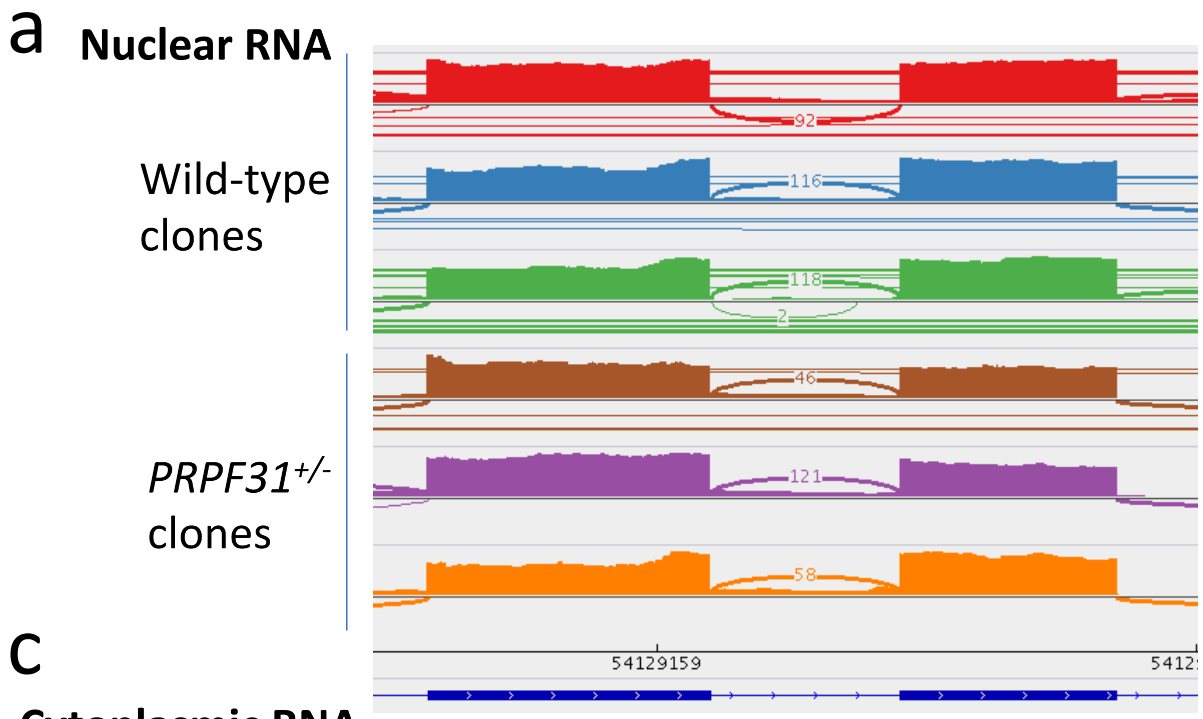

b

	PValue	FDR	Intron inclusion level, WT	Intron inclusion level, *PRPF31*+/- clones	Intron inclusion level difference
SJ	0.00011932	0.01405932	0.096,0.09,0.039	0.132,0.186,0.194	-0.096
SJ+I	0.0001194	0.00906623	0.096,0.09,0.038	0.132,0.185,0.193	-0.095

c

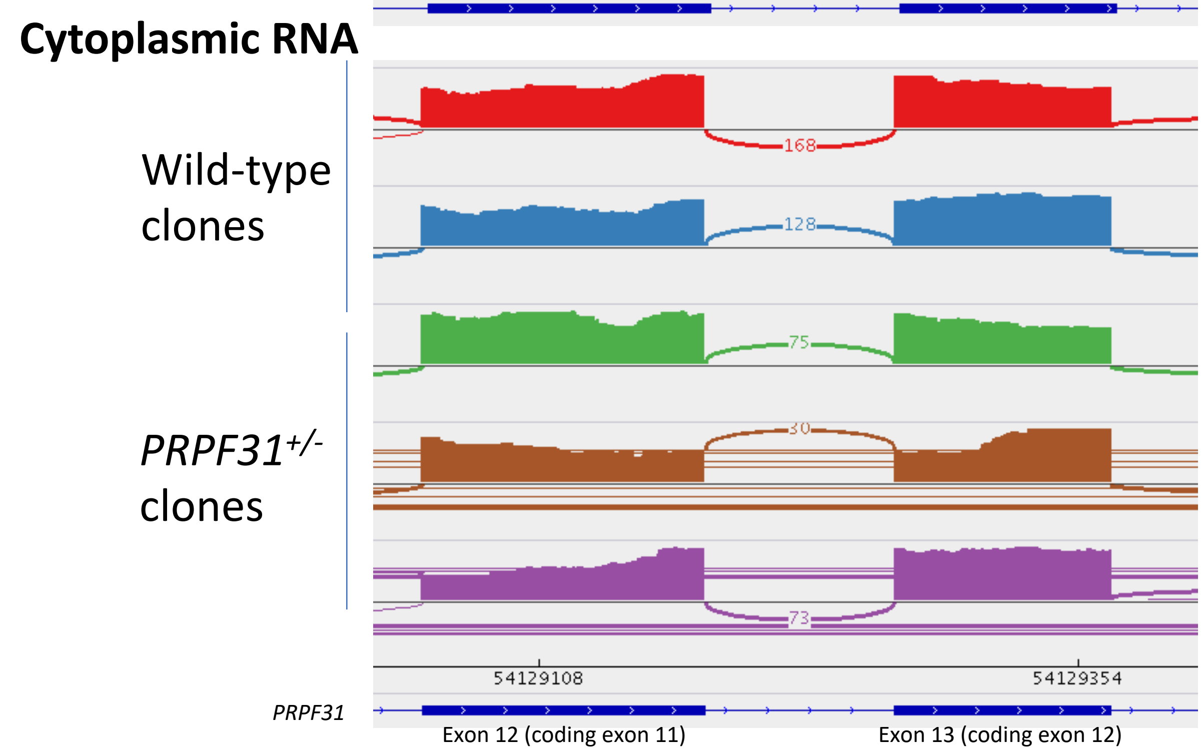

d

	PValue	FDR	Intron inclusion level, WT	Intron inclusion level, *PRPF31*+/- clones	Intron inclusion level difference
SJ	1	1	0.004,0.0	0.009,0.0,0.0	-0.001
SJ+I	1	1	0.004,0.0	0.009,0.0,0.0	-0.001

SJ = only reads mapping to splice junctions considered
SJ + I = reads mapping to splice junctions and to intron considered
