# Supplementary material for: A CRISPR and high-content imaging assay compliant with ACMG/AMP guidelines for clinical variant interpretation in ciliopathies
Source: Hum Genet. 2020 Oct 23;140(4):593–607. doi: 10.1007/s00439-020-02228-1 (PMC7981318; doi:10.1007/s00439-020-02228-1)
Supplement: Supplementary file 2 — Supplementary file2 Supplementary Figure 2 – High content image analysis workflow of nuclei and cilia in wild-type and PRPF31+/- mutant clones. (a) Top two rows of panels show DAPI stained nuclei and ARL13B antibody-stained cilia from wild-type and mutant cells in raw output images from Opera confocal high-throughput imager. Lower four panels show automated image analysis using CellProfiler. Insets show magnified images from each panel. (PDF 1745 kb) [file 439_2020_2228_MOESM2_ESM.pdf]

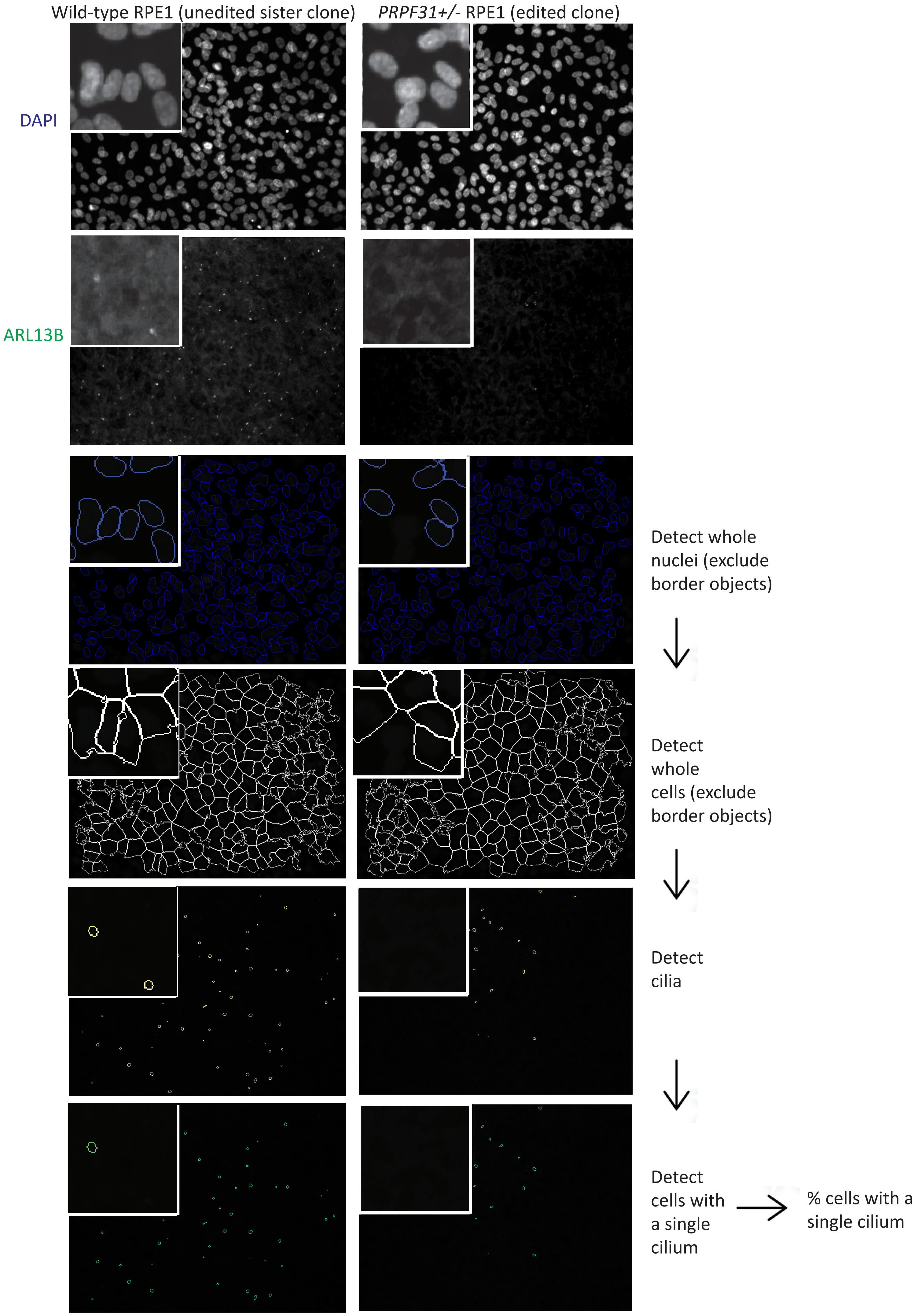
Wild-type RPE1 (unedited sister clone)
PRPF31+/- RPE1 (edited clone)
DAPI
ARL13B
Detect whole nuclei (exclude border objects)
Detect whole cells (exclude border objects)
Detect cilia
Detect cells with a single cilium
% cells with a single cilium
